# Principal component analysis fosr fast and model-free denoising of multi *b*-value diffusion-weighted MR images

**DOI:** 10.1088/1361-6560/ab1786

**Published:** 2019-05-16

**Authors:** Oliver J Gurney-Champion, David J Collins, Andreas Wetscherek, Mihaela Rata, Remy Klaassen, Hanneke W M van Laarhoven, Kevin J Harrington, Uwe Oelfke, Matthew R Orton

**Affiliations:** 1 Joint Department of Physics, The Institute of Cancer Research and The Royal Marsden NHS Foundation Trust, London, United Kingdom; 2 Cancer Research UK Cancer Imaging Centre, The Institute of Cancer Research and The Royal Marsden NHS Foundation Trust, London, United Kingdom; 3 Department of Medical Oncology, Cancer Center Amsterdam, Amsterdam UMC, University of Amsterdam, Amsterdam, The Netherlands; 4 Targeted Therapy Team, The Institute of Cancer Research and The Royal Marsden NHS Foundation Trust, London, United Kingdom; 5 Laboratory for Experimental Oncology and Radiobiology, Center for Experimental and Molecular Medicine, Cancer Center Amsterdam, Amsterdam UMC, University of Amsterdam, The Netherlands; oliver.gurney-champion@icr.ac.uk

**Keywords:** diffusion-weighted MRI, denoising, intravoxel incoherent motion, principal component analysis, synthetic MRI, motion

## Abstract

Despite the utility of tumour characterisation using quantitative parameter maps from multi-*b*-value diffusion-weighted MRI (DWI), clinicians often prefer the use of the image with highest diffusion-weighting (*b*-value), for instance for defining regions of interest (ROIs). However, these images are typically degraded by noise, as they do not utilize the information from the full acquisition. We present a principal component analysis (PCA) approach for model-free denoising of DWI data. PCA-denoising was compared to synthetic MRI, where a diffusion model is fitted for each voxel and a denoised image at a given *b*-value is generated from the model fit. A quantitative comparison of systematic and random errors was performed on data simulated using several diffusion models (mono-exponential, bi-exponential, stretched-exponential and kurtosis). A qualitative visual comparison was also performed for *in vivo* images in six healthy volunteers and three pancreatic cancer patients. In simulations, the reduction in random errors from PCA-denoising was substantial (up to 55%) and similar to synthetic MRI (up to 53%). Model-based synthetic MRI denoising resulted in substantial (up to 29% of signal) systematic errors, whereas PCA-denoising was able to denoise without introducing systematic errors (less than 2%). *In vivo*, the signal-to-noise ratio (SNR) and sharpness of PCA-denoised images were superior to synthetic MRI, resulting in clearer tumour boundaries. In the presence of motion, PCA-denoising did not cause image blurring, unlike image averaging or synthetic MRI. Multi-*b*-value MRI can be denoised model-free with our PCA-denoising strategy that reduces noise to a level similar to synthetic MRI, but without introducing systematic errors associated with the synthetic MRI method.

## 1. Introduction

Diffusion-weighted (DW) MRI (DWI) is an important tool for diagnosis (Koh *et al* 2007, 2011, Barral *et al* 2015), treatment response monitoring (Koh and Collins 2007, Park *et al* 2014, Barral *et al* 2015, Klaassen *et al* 2018) and radiotherapy treatment planning (Gurney-Champion *et al* 2017, Heerkens *et al* 2017). For DWI there is a trend to acquire images at multiple diffusion-weightings (*b*-values) that are used to generate quantitative metrics by fitting signal models, such as the intravoxel incoherent motion (IVIM), kurtosis and stretched-exponential models (Le Bihan *et al* 1988, Jensen *et al* 2005, Bennett *et al* 2003). These models, in turn, allow more specific tissue characterisation than the classically used apparent diffusion coefficient (ADC) retrieved from a mono-exponential model; for example, the bi-exponential IVIM model gives additional information on tissue perfusion (Le Bihan *et al* 1988).

Despite the utility of tumour characterisation using quantitative maps from model fitting, clinicians often prefer assessing images and defining regions of interest (ROIs) directly on high *b*-value DW-images (Koh and Collins 2007) because the contrast-to-noise ratio (CNR) of the lesion is optimal in such images. However, high *b*-value images often have poor signal-to-noise ratio (SNR), and only utilize a fraction of the information from the DWI acquisition, as measurement time is divided among all *b*-values. For example, it was recently suggested that a minimum of 16 *b*-values are desired for IVIM in the liver (ter Voert *et al* 2016), resulting in only 6% of acquisition time spent at acquiring a particular *b*-value. One way of dealing with poor SNR is to directly denoise the individual images, which is often done using a smoothing kernel (Manjón *et al* 2010, 2013, 2015, Bustin *et al* 2017), such as a Gaussian filter. Such approaches use spatial information to denoise the image and hence lead to undesirable image blurring.

**Figure 1.**
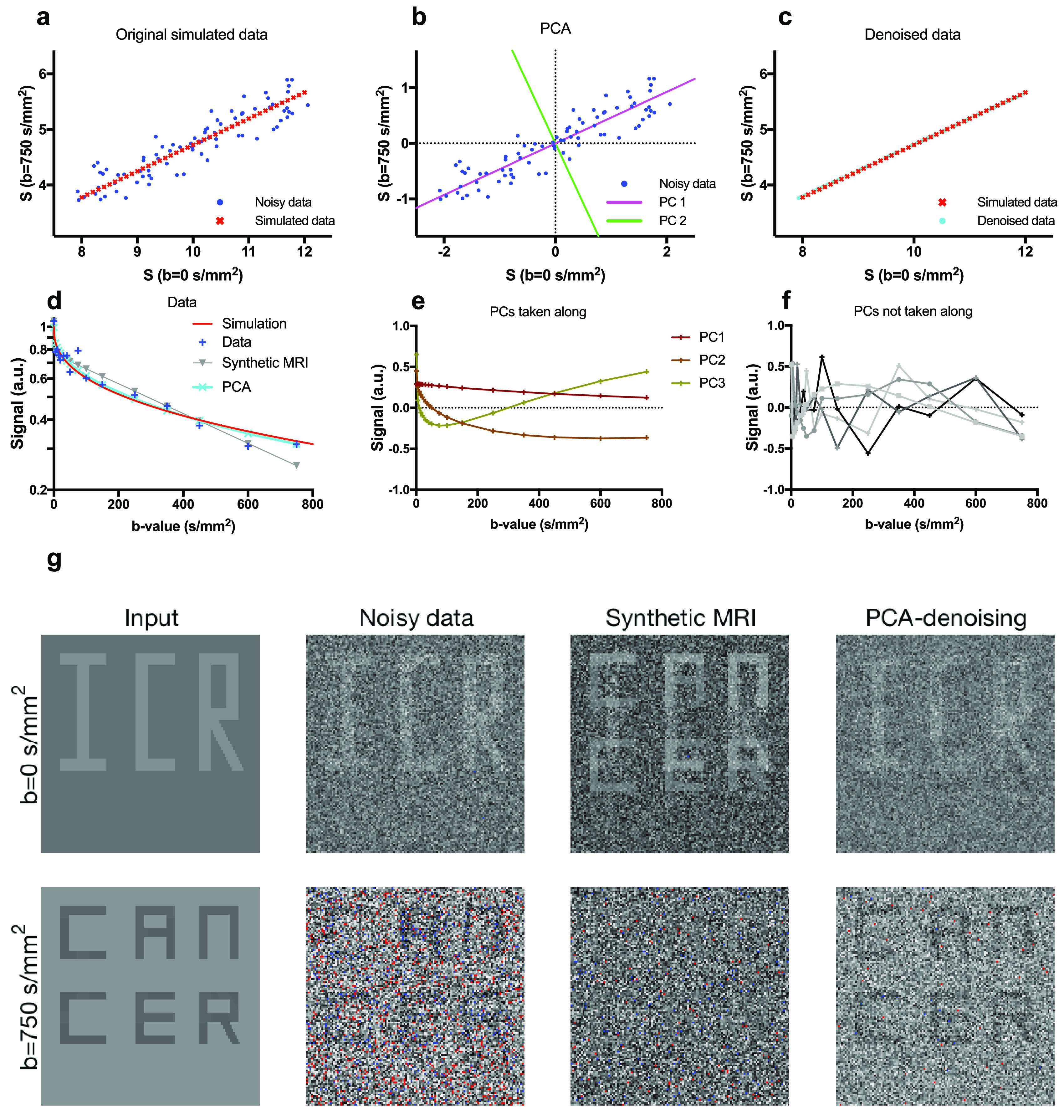
Panels (a)–(c) show a simplified example of PCA-denoising with only two *b*-values. Panels (d)–(g) show synthetic MRI and PCA-denoising on data simulated using the stretched-exponential model. In this example, synthetic MRI produced a systematic error that obscured ‘cancer’ on the high *b*-value image, whereas PCA-denoising did not. Details of this example are discussed in the main text. In panel (g), voxels with values outside the window level setting range are red (upper limit) and blue (lower limit).

Alternatively, for DWI, information from all the *b*-value images can be used to enhance the SNR in the high *b*-value image. This has previously been achieved using synthetic MRI (also known as computed DWI) (Bobman *et al* 1985, Blackledge *et al* 2011). In synthetic MRI a diffusion model (typically mono-exponential) is fitted as a function of *b*-value in each voxel to yield corresponding parameter maps. A synthetic image for a given *b*-value is then generated using the parameters from the fit. This technique certainly enhances the SNR of the image but has the disadvantage that synthetic MRI only considers effects that are described by the model. Systematic errors (i.e. bias) will occur when the underlying signal-decay is different from that described by the model, potentially obscuring relevant pathology, as shown using simulated data in figure 1(g). Furthermore, when more complex models and fitting algorithms are used, the computation time of synthetic MRI can be prohibitive.

To prevent these systematic errors, a denoising approach that does not use a model is desirable since it has the potential to work well for a wide range of tissue properties, including those outside the scope of currently available signal models. We believe principal component analysis (PCA) is a promising candidate for denoising as it is fast, has the potential to reduce motion between individual images (Melbourne *et al* 2007), and is model-free. In the past, PCA has been applied mainly in neuroimaging in several approaches, including anatomical imaging (spatial domain, using voxels from the same image) (Muresan and Parks 2003, Manjón *et al* 2015), and quantitative imaging (quantitative domain; i.e. same voxel with repeated measures such as DWI) (Bydder and Du 2006, Balvay *et al* 2011, Manjón *et al* 2013). We set out to develop an easily implementable PCA-denoising approach for improving image quality of IVIM images that could be applied in any anatomy, including free-breathing abdomen images.

In this paper, we show that PCA can be used for model-free denoising of multi-*b*-value DWI data, and we compare its statistical properties to synthetic MRI using a range of DWI models.

## 2. Materials and methods

All image processing was done using MATLAB (2018a, The MathWorks, Inc., Natick, Massachusetts) and plots were created with GraphPad Prism (Version 7.0c for Mac, GraphPad Software, San Diego, California). The PCA-denoising MATLAB script can be found in Supplemental Digital Content 1 (stacks.iop.org/PMB/64/105015/mmedia).

### 2.1. PCA-denoising

PCA separates potentially correlated observations into linearly uncorrelated orthogonal vectors, called principal components (PCs) (Hotelling 1933). After centering the data (subtracting the overall mean), the first PC contains the most variation, and each subsequent PC contains the largest remaining variation. In our proposed PCA-denoising approach, after subtracting the mean signal decay, PCA was applied to the signal vectors from each voxel of a DWI-dataset, where the vector elements in each voxel correspond to each of the acquired *b*-values and gradient directions. The number of vectors used to compute the PCs hence corresponds to the number of voxels, whereas the length of each vector corresponds to the number of acquisitions (*b*-values, directions, averages). PCA also returns the corresponding vectors of weights per PC (called scores), and the sum of all PCs multiplied by their matching scores and addition of the mean signal decay then returns the original signal from each voxel:

1 \begin{align*} \newcommand{\e}{{\rm e}} \displaystyle {{{S}}_{j}}={m}+{{{T}}_{j}}\cdot {{{W}}^{T}},\nonumber \end{align*}

where *S*_*j* _ is a row vector containing the reconstructed denoised attenuation curve for voxel *j* , *m* is the mean signal per measurement (over all voxels), *T*_*j* _ is the score vector (row vector) for voxel *j*  and *W* is an orthonormal matrix whose columns are the PC vectors. The informative signal decay appears in earlier PCs because the variation in these signals tends to be correlated across voxels and have large variation (corresponding to genuine tissue/image contrast), whereas the later PCs correspond to noise since they are uncorrelated across voxels and have lower variation. By reconstructing the DW-images from only the initial informative PCs, multiplied by their corresponding PC scores per voxel, a denoised DWI dataset can be generated. In equation (1) this denoising is performed by setting the elements of *T*_*j* _ that correspond to the rejected PCs to zero.

In summary:

• DWI image volumes from all acquired *b*-values, diffusion directions and averages are reformatted into an *N*  ×  *M* data matrix of *N* voxels and *M* measurements (*b*-values  ×  diffusion directions  ×  averages).
• PCA is performed over the rows of the data matrix (described in section 2.1.1), returning *M* PCs (*M*  ×  *M* matrix *W* in equation (1)), as well as their scores per voxel (*N*  ×  *M* matrix whose *j* th row is *T*_*j* _ for voxels *j*   =  1, 2, …, *N*).
• The informative PCs that should be included in the reconstruction of the denoised images are selected using the methods described in section 2.1.2.
• Finally, a denoised image is reconstructed using only the selected PCs by zeroing the corresponding elements of the score vectors in equation (1).

#### 2.1.1. Simplified PCA-denoising

Figures 1(a)–(c) illustrate a simplified example of PCA-denoising from data that was simulated with a mono-exponential model with only two *b*-values (*b*  =  0 and 750 s mm^−2^). In this example, the baseline (*b*  =  0 s mm^−2^) signal magnitude of the model varied across data points, while the ADC was fixed. This implies that the noiseless signal data, shown by the red dots in panel 1(a), have only one degree of freedom. After subtracting the mean signal, PCA resulted in two PCs (figure 1(b)). By reconstructing the signal with only the first PC, the noise was substantially reduced (figure 1(c)).

Figures 1(d)–(g) show an example with sixteen *b*-values, in the range 0–750 s mm^−2^, that was designed to illustrate the drawback of the model dependence of synthetic MRI compared to PCA-denoising. In this example, data were simulated by a stretched-exponential with parameters per voxel chosen from a clinically relevant range such that the letters ‘ICR’ appeared on the *b*  =  0 s mm^−2^ image and ‘cancer’ on the *b*  =  750 s mm^−2^ image (stretched exponential model parameters described in table S1; *D*  =  1.8, 1.625, 1.675 and 1.96  ×  10^–3^ s mm^−2^; *α*  =  0.4, 1, 1, 0.4; *S*0  =  1, 1, 1.04, 10.4). The synthetic MRI was generated by a bi-exponential fit. The discrepancy between the models reflects real-life denoising in which the true signal decay is typically unknown and is not fully described by the available models used for denoising. An example voxel from the background region in figure 1(g) is shown in figure 1(d). For this voxel, the bi-exponential fit underestimates the signal at high *b*-values, which ultimately causes the contrast between background and ‘cancer’ to vanish in figure 1(g), whereas PCA gives an accurate estimation of the signal intensity. The three retained PCs that were included in the denoised image and the initial five excluded PCs are illustrated in figures 1(e) and (f). It is clear that PCA was able to reduce noise while retaining the contrast and visibility of the relevant pathology (‘cancer’ and ‘ICR’), whereas in synthetic MRI the contrast between cancer and background at *b*  =  750 s mm^−2^ was removed.

#### 2.1.2. Robust PCA-denoising

To denoise DWI datasets, the informative PCs derived from the signal decay need to be separated from the PCs that contain mainly noise. For this purpose, we developed a two-step approach. The first step aims at identifying the PCs that contain at least 97% of the signal information. To achieve this, first, the information contained in each PC was estimated from the PC scores ***T***_*j*_ (see equation (1)) using the following equation

**Figure 2.**
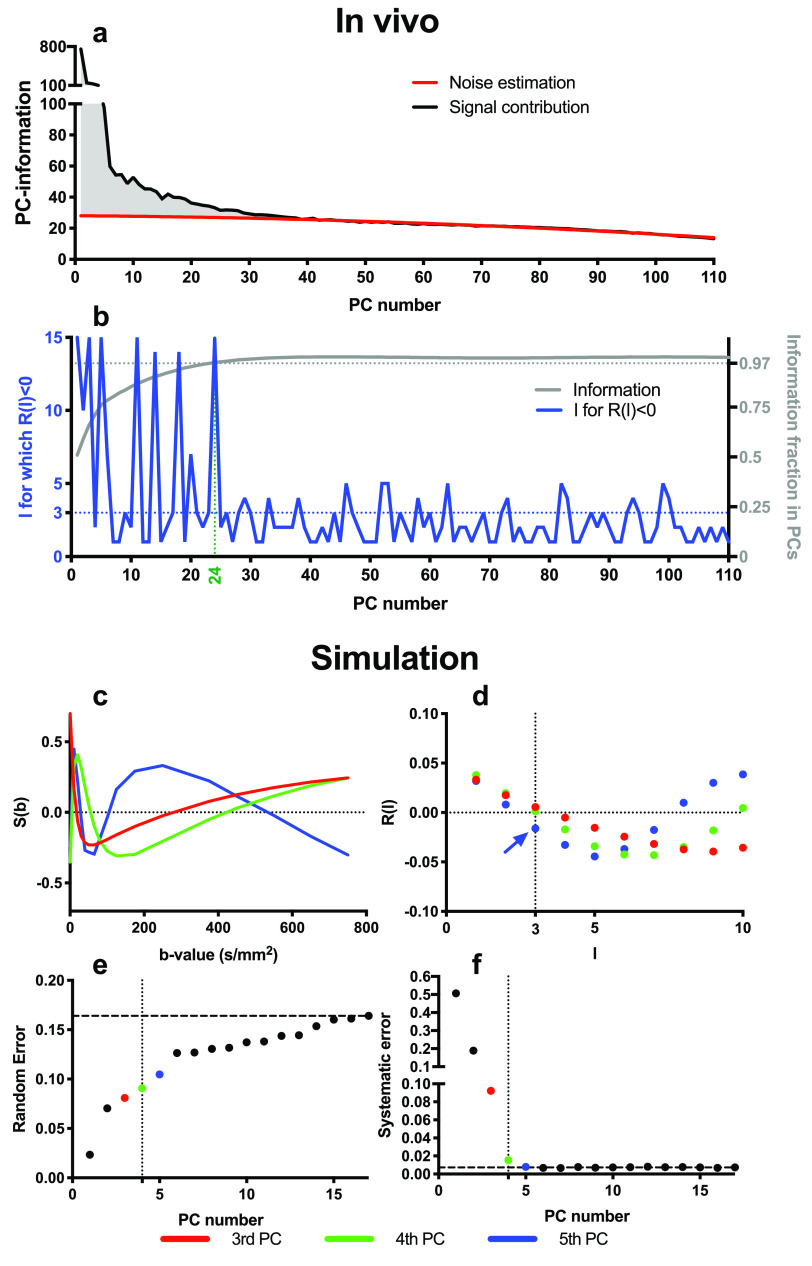
Panel (a) illustrates the initial selection procedure from *in vivo* data which guaranteed 97% of all information was included (see main text for details). Panel (b) then illustrates that for this patient, the l for which *R*(*l*)  <  0 of the next PC also was below 3, so 24 PCs were selected. Panels (c)–(f) show an example where four PCs were determined to be the optimal number to retain in this example of bi-exponential simulated data (SNR  =  20). Panel (c) shows the last two retained PCs: 3rd (red) and 4th (green), and the first rejected PC: 5th (blue). Panel (d) shows the autocorrelation function estimates (points). The blue arrow highlights the first PC with *R*(*l*  ⩽  3)  <  0. Panels (e) and (f) show, respectively, the random and systematic errors as a function of the number of PCs taken along. The horizontal dashed lines in (c) and (d) show the corresponding random and systematic errors to the noisy data.

2 \begin{align*} \newcommand{\e}{{\rm e}} \displaystyle {{M}_{PC,m}}=\frac{\mathop{\sum }_{j=1}^{N}\left| {{\boldsymbol{T}}_{j,m}} \right|}{N},\nonumber \end{align*}

where ***T***_*j* ,*m*_ is the *m*th element of the score vector for voxel *j* , and *M*_*PC*,*m*_ is the ‘PC-information’ of the *m*th PC. Figure 2(a) plots the PC-information as a function of PC index (black line) from *in vivo* data and the pattern shown, in particular the rapid decay followed by a slow roll-off in the latter PCs, is typical for *in vivo* data. In theory, for a sufficiently large dataset, normally distributed noise would give a constant offset in the PC-information of all PCs, so subtracting this from the plot would hence give the signal contribution to the PC-information per PC. However, due to the dataset being finite, and PCs being sorted according to their variance, the noise contribution gradually decreases as function of PC index. To account for this, we assume that the last 2/3 of the PCs only describe noise, and fit a second order polynomial to these PCs to estimate the noise contribution per PC. The signal contribution to the PC-information is then given by the distance between the estimated noise contribution (red line) and the overall PC-information (black line), and the cumulative sum of this adjusted curve up to a given PC represents the total information included in the PCs up to that PC. This adjusted cumulative sum, normalized by the sum over all PCs, is plotted in grey in figure 2(b) and was used to determine a threshold where at least 97% of the informative signal was retained.

In simulated data, we found this criterion alone still excluded several apparently informative PCs, and hence a second step was added to include these PCs. In this step, for each of the PCs we calculated the autocorrelation function (figure 2(d) for simulated example and figure 2(b) for *in vivo* example), defined as

3 \begin{align*} \newcommand{\e}{{\rm e}} \displaystyle {{R}_{j}}(l)=\frac{1}{(M-l){{\sigma }_{j}}^{2}}\underset{i=1}{\overset{M-l}{\mathop \sum }}\,\left( {{W}_{i,j}}-{{{\bar{W}}}_{j}} \right)\times \left( {{W}_{i+l,j}}-{{{\bar{W}}}_{j}} \right),\nonumber \end{align*}

for delays *l*  =  1, 2, …, *M*  −  1, where *W*_*i*,*j*_ are the elements of the *j* th PC being tested, and ${{\bar{W}}_{j}}$ and ${{\sigma }_{j}}$ are the mean and standard deviation of *W*_*i*,*j* _ over the *j* th PC. We have empirically found in simulations that PCs with positive autocorrelation for small delays correspond to genuine diffusion effects, whereas PCs with one or more negative autocorrelation values for small delays correspond to noise. This effect can be detected by identifying PCs where *R*(*l*)  >  0 for all *l*  ⩽  *L*, and we have found that *L*  =  3 is an appropriate threshold in this application. Therefore, for PCs above the 97% threshold, subsequent PCs were also included up to the first PC that failed the autocorrelation criteria. The first PC that failed the autocorrelation criteria and all subsequent PCs were discarded as noise and removed during reconstruction of the denoised images. Figures 2(c)–(f) show in simulated data how this approach selected the first four PCs for reconstructing the denoised images, resulting in a favourable trade-off between random and systematic errors.

Since the autocorrelation function is affected by the ordering of the data (*b*-values and directions), for *in vivo* data, *R*(*l*) was not only calculated for data ordered by *b*-value but also for data grouped by diffusion direction; only when both autocorrelation (*R*_1_ and *R*_2_) functions fulfil *R*(*l*  ⩽  3)  <  0 was the PC classified as non-informative. In the *in vivo* example (figures 2(a) and (b)), 24 PCs were selected (green vertical line).

### 2.2. Synthetic MRI denoising

For comparison with PCA-denoising, synthetic MRI denoising using mono-exponential, bi-exponential stretched-exponential and kurtosis models was performed with linear least squares fits.

### 2.3. Simulations

**Figure 3.**
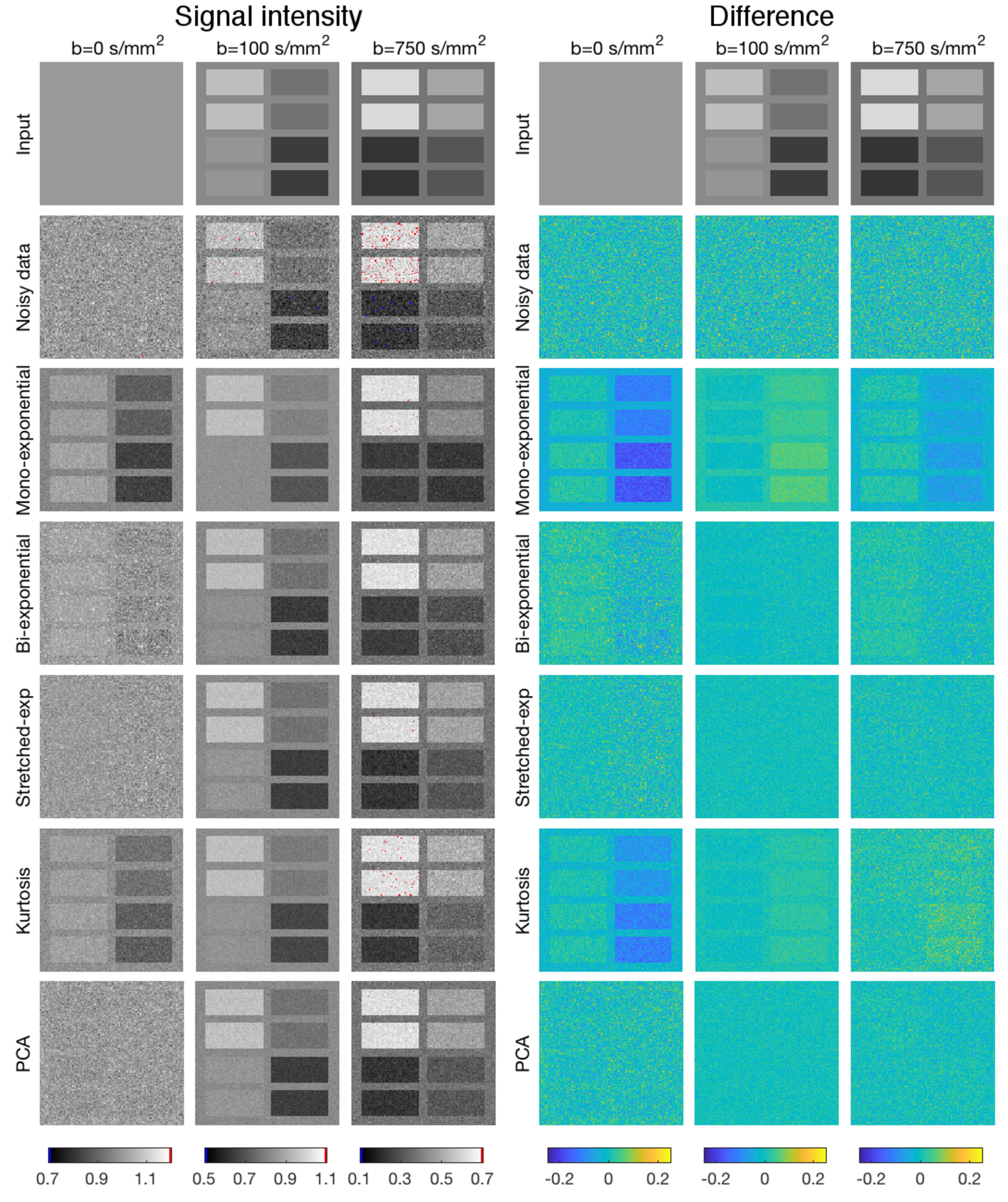
Left panel: Simulated stretched-exponential images with SNR  =  20, showing *b*  =  0, 100 and 750 s mm^−2^ (left to right), noisy data; mono-exponential, bi-exponential, stretched-exponential and kurtosis synthetic MRI; and PCA-denoising (top to bottom). Voxels with values outside the grey level range are red (upper limit) and blue (lower limit). Right panel: Difference in signal between the input and the denoised images, similar layout to the left panel. Mono-exponential and bi-exponential synthetic MRI introduced systematic errors for some, or all *b*-values, which resulted in some sub-panels appearing above the noise in the difference image. Testing panel configuration based on While (2017).

We compared our PCA-denoising approach with synthetic MRI by applying both to training data and test images that were generated from mono-exponential (classic ADC), bi-exponential (IVIM) (Le Bihan 1988), stretched-exponential (Bennett *et al* 2003) and kurtosis (Jensen *et al* 2005) signal decay models with 17 *b*-values (0, 10, 20, 30, 40, 50, 65, 80, 100, 125, 175, 250, 375, 450, 550, 650 and 750 s mm^−2^). Test images of 128  ×  128 voxels consisting of eight 23  ×  51 voxel subpanels were generated (figure 3) enabling nine regions of distinct simulation parameters (While 2017). The model parameters for the eight subpanels were set to combinations of the values given in table S1 from Supplemental Digital Content 2, giving eight combinations for the bi-exponential model, four (repeated twice) for the stretched-exponential and kurtosis models, and two (repeated four times) for the mono-exponential model. Gaussian noise was added to the data to simulate SNRs of 10, 20, 50 and 80 at *b*  =  0 s mm^−2^. The SNR range reflects SNR typically seen in DWI, with the low values typical for anatomy with poor SNR (i.e. liver/pancreas with short T2) and individual images, whereas the high values are seen in anatomy with high SNR (i.e. brain, with long T2) and when repeated data are averaged.

PCs were then determined using both the test (3.5%) and training (96.5%) data, and the PCs selected using the autocorrelation method described above were used to denoise only the 128  ×  128 test images.

Since the 128  ×  128 test images consisted of panels of similar behaviour, it was possible to estimate both systematic and random errors. The random error was defined as the standard deviation over the signal within each sub-panel and overall random error was then defined as the root-mean-square of this. The systematic error (i.e. bias) was defined as the difference between the mean denoised signal per panel and the true noiseless signal. The maximum systematic error from the nine regions was then reported.

### 2.4. *In vivo* evaluation

Local ethics committee approval was granted for healthy (at the ICR/RMH) and patient (at the Amsterdam UMC) volunteer scans, and all patient and healthy volunteers gave written informed consent.

DWI datasets from five healthy volunteers were obtained at the ICR/RMH on two different 1.5 T scanners (Aera and Avanto; Siemens Healthineers, Erlangen, Germany). To illustrate the flexibility of the denoising approach, each volunteer underwent a different protocol in different anatomical regions and data consisted of an axial leg, three abdominal scans (two axial, one coronal) and a brain scan. Furthermore, the brain dataset from Peterson (2016) (https://doi.org/10.6084/m9.figshare.3395704.v1), an online dataset of which we could not trace the acquisition parameters other than the *b*-values, was also analysed to show the flexibility of PCA-denoising.

DWI datasets from three pancreatic cancer patients were obtained at the AMC on a 3T scanner (Ingenia; Philips Healthcare, Best, The Netherlands). These patient data have been previously published (Gurney-Champion *et al* 2018, Klaassen *et al* 2018) for the purpose of testing the performance of fit algorithms and different diffusion models (without PCA-denoising). In our current work, they serve as an example of how PCA-denoising works in patient data.

Acquisitions were done using 2D multi-slice DW echo-planar imaging (table 1). All images were denoised using PCA-denoising. *In vivo* data requires additional pre-processing to remove background pixels that are outside the anatomy and therefore contain only noise. These voxels were identified using a manually chosen threshold on the mean *b*  =  0 s mm^−2^ image, and were excluded in PCA-denoising. As the bi-exponential synthetic MRI approach gave the least systematic errors in the simulated data, we denoised the *in vivo* dataset with bi-exponential synthetic MRI as comparison.

**Table 1.** MRI settings.

	Volunteer					Patient
Region	Leg	Abdomen axial/ axial #2^a^	Abdomen coronal	Brain	Brain #2 (Peterson 2016)	Pancreatic cancer
Scanner	Aera (1.5 T, Siemens)	Avanto (1.5 T, Siemens)	Avanto (1.5 T, Siemens)	Avanto (1.5 T, Siemens)	Unknown	Ingenia (3T, Philips)
Orientation	Axial	Axial	Coronal	Axial	Axial	Axial
Resolution (mm^2^)	2.4 × 2.4	2.7 × 2.7	1.6 × 1.6	2.3 × 2.3	0.94 × 0.94	3.0 × 3.0
FOV (mm^2^)	188 × 400	284 × 350	400 × 400	243 × 300	240 × 240	432 × 108
Slice thickness (mm)	5.0	8.0	5.0	8.0	2.5	3.7
Slice gap (mm)	0.0	0.8	0.0	8.0	0.0	0.3
Slices	25	8	20	8	54	18
TR/TE (ms)	4800/58	1300^b^/113	5000/60	2900/113	Unknown	2200^b^/45
Respiratory compensation	—	Triggered (bellow)	None	—	—	Triggered (navigator)
*b*-values (s mm^−2^)	0, 10, 20, 25, 40, 50, 75, 100, 300, 500, 700, 900	0, 2, 7, 10, 20, 30, 40, 50, 65, 85, 110, 150, 250, 350, 450, (650, 900)^a^	0, 20, 40, 60, 120, 240, 480, 900	0, 2, 7, 10, 20, 30, 40, 50, 65, 85, 110, 150, 250, 350, 450, 650, 900	0, 10, 20, 30, 40, 60, 80, 100, 120, 140, 160, 180, 200, 300, 400, 500, 600, 700, 800, 900, 1000	0, 10, 20, 30, 40, 50, 75, 100, 150, 250, 400, 600
Directions	6	6	3	6	Trace image (3 directions)	15, 9, 9, 9, 9, 9, 4, 12, 4, 4, 16
Averages	*b* < 75 → 1	1	5	1	Unknown	1
	*b* ⩾ 75 → 2					

CPU time (s)		Axial/axial#2				Mean (range)
PCA-denoising	1.3	0.41/0.46	7.3	0.37	21.1	0.74 (0.67–0.86)
Mono-exponential	67.0	12.3/19.0	289.8	13.6	626.3	28.6 (24.5–33.8)
Bi-exponential	196.9	29.6/54.2	937.3	26.5	1355.2	80.8 (70.1–92.1)

a Both volunteers were scanned with the same protocol, except axial #2 included a 8 mm slice gap and *b*  =  650 and 900 s mm^−2^.

b True echo time depends on respiratory triggering. 1300 ms (volunteer) and 2200 ms (patient) were the minimum repetition times set.

## 3. Results

### 3.1. Simulations

Figure 3 shows an example of how PCA-denoising reduced the noise without creating a systematic error, whereas synthetic MRI using the non-matching models caused pronounced systematic errors in this simulated stretched-exponential data.

**Figure 4.**
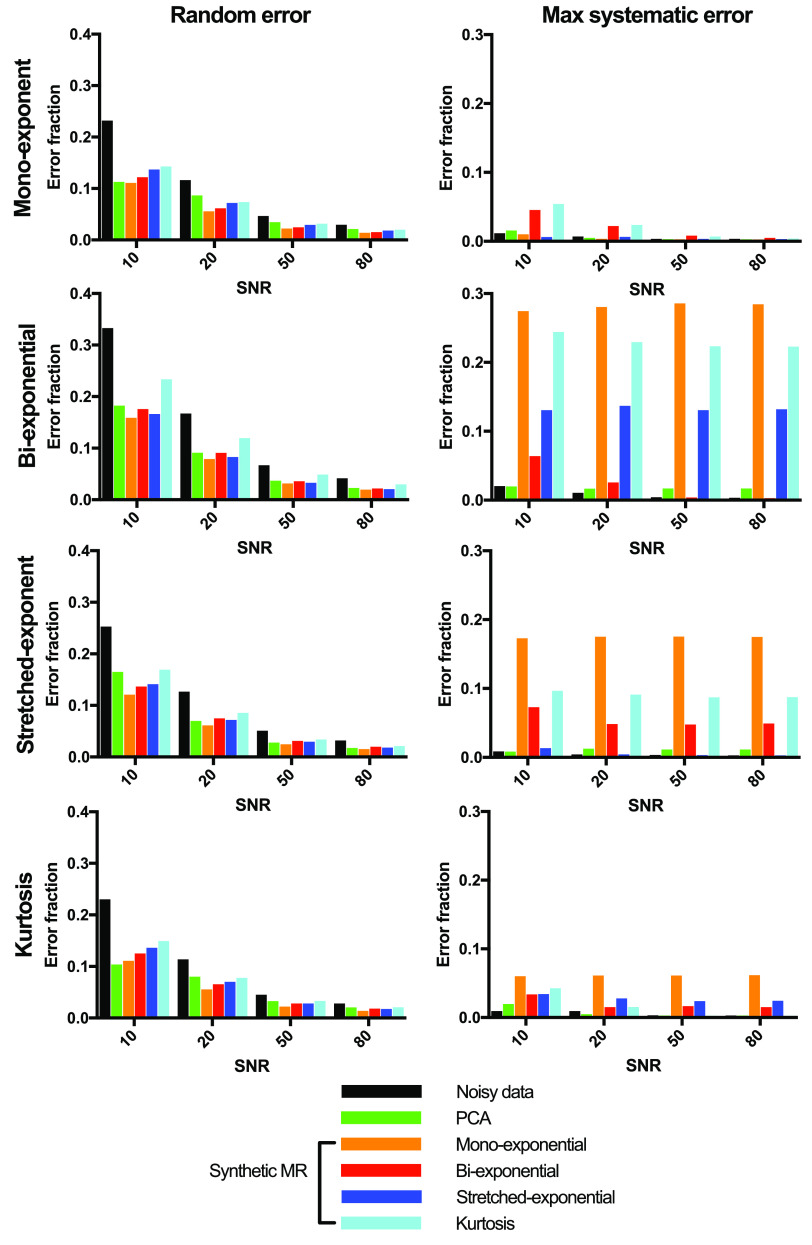
Random (left) and systematic (right) error fractions (error normalised to total signal intensity) from simulated noisy data (black) and denoised data using either PCA (green) or synthetic MRI (orange, red, blue, cyan) in the highest *b*-values image (750 s mm^−2^). The change in signal fraction (=magnitude of error divided by total signal) is plotted as a function of SNR of the simulated data. The root-mean-square of the random error maximum systematic error from the nine regions is shown.

**Figure 5.**
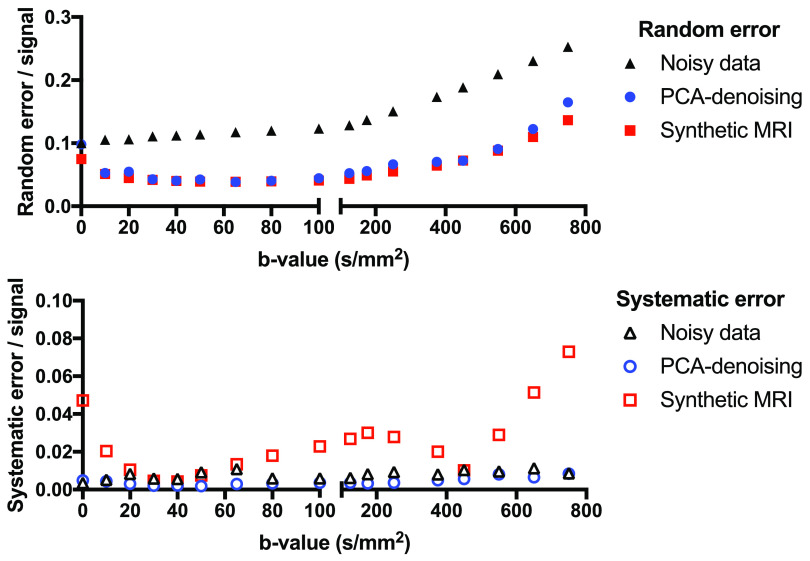
Plot of the performance of PCA-denoising over all *b*-values of simulated stretched-exponential data with SNR  =  10. Synthetic MRI using a bi-exponential model is plotted as a reference.

All denoising approaches decreased the random error, whereas only PCA was capable of doing this for every dataset without introducing systematic error (figure 4). The difference images in figure 3 and the plots in figure 5 show that PCA reduced the noise most effectively for intermediate *b*-values, but was also effective at high *b*-values. For high *b*-values, PCA-denoising decreased the random noise by up to 55%, while the maximum systematic error was less than 2% (figure 4). For the synthetic MRI approaches, the maximum decreases in random errors were 53%, 48%, 51% and 38%, whereas the maximum systematic errors were 29%, 7%, 14% and 24% for the mono-exponential, bi-exponential, stretched-exponential and kurtosis models, respectively. Two to four PCs were selected for denoising, with generally more PCs selected at higher SNR levels and for data simulated by models with more parameters.

### 3.2. *In vivo* evaluation

**Figure 6.**
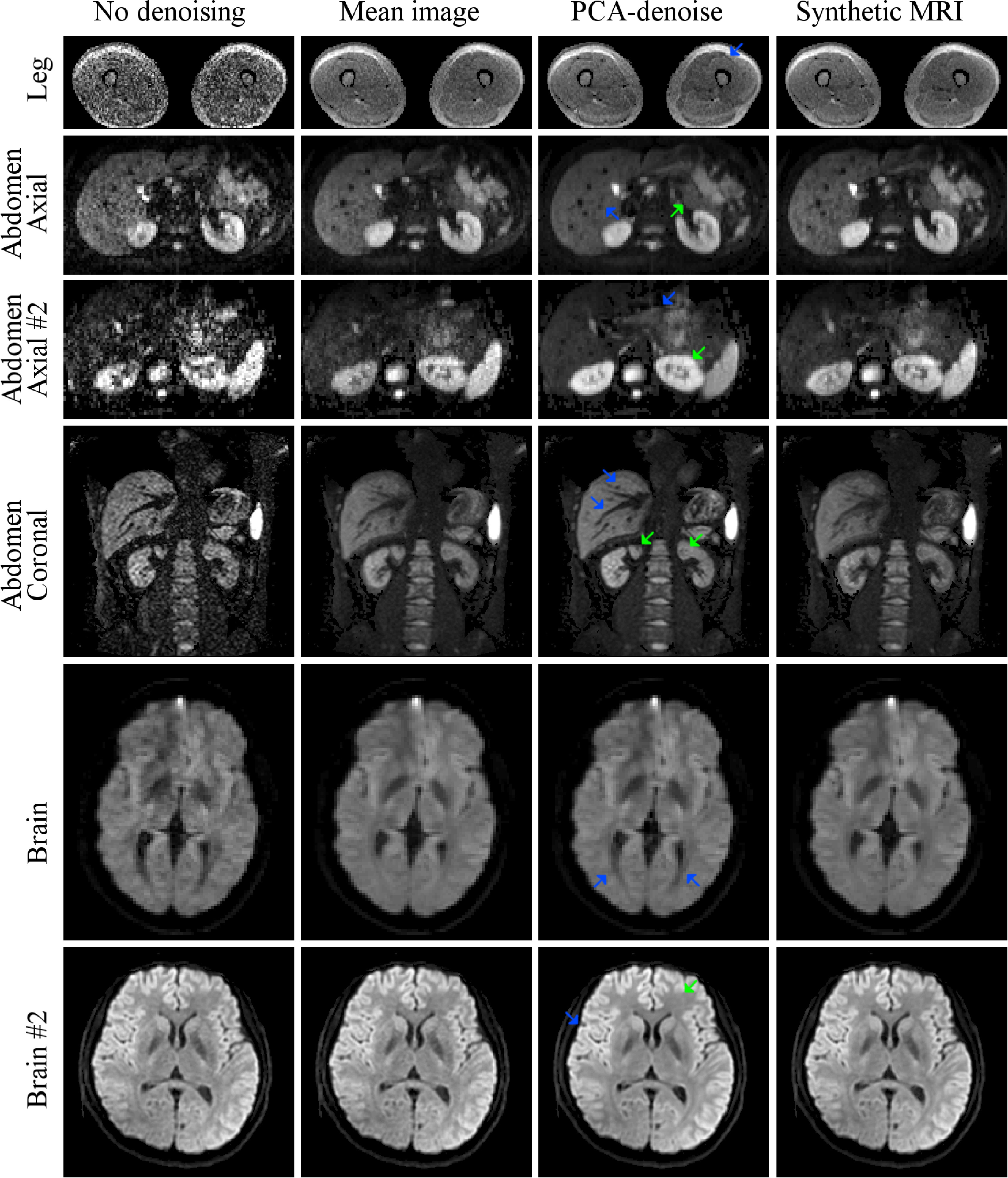
*In vivo* example of PCA-denoising (3rd column), compared to mean image over all repeated measures (2nd column) and bi-exponential synthetic MRI (4th column). All show the highest *b*-value image from the series, except abdomen axial #2, which shows the second highest *b*-value *b*  =  650 s mm^−2^ as no signal was left at the highest *b*-value due to the long TE (113 ms) combined with short T2-times in the abdomen. Arrows are discussed in the main text.

**Figure 7.**
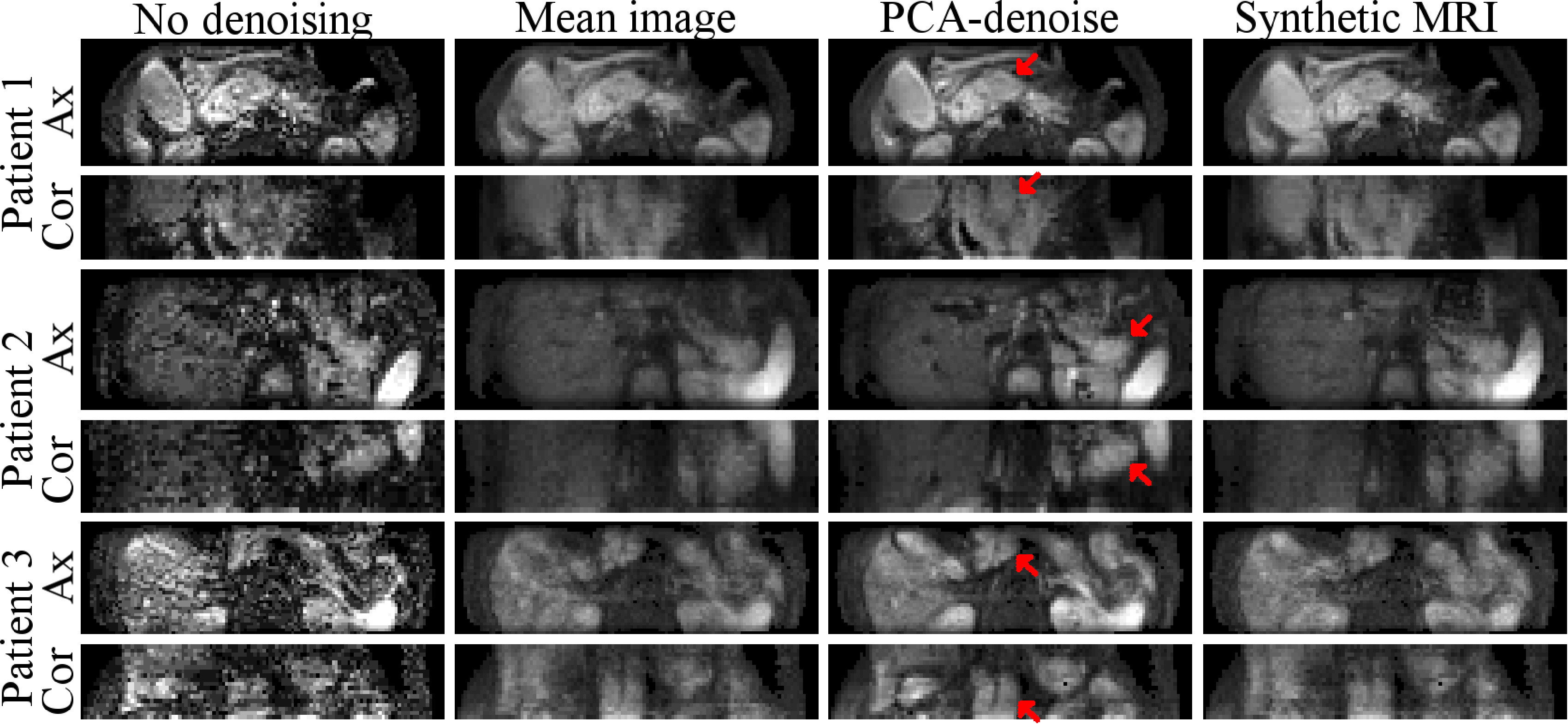
Axial slices and coronal reconstructions of the *b*  =  600 s mm^−2^ image from pancreatic cancer patients after PCA-denoising (3rd column) compared to the original data (1st column), averaging (2nd column) and bi-exponential synthetic MRI (4th column). Red arrows indicate tumour locations. Compared to PCA-denoising, the other approaches blur the image (mainly visible in the coronal reconstruction), preventing accurate boundary detection.

*In vivo*, PCA-denoising decreased the noise at least as effectively as synthetic MRI for different scanners, sequences and anatomical regions (figures 6 and 7). The systematic error introduced by synthetic MRI cannot be quantified *in vivo*, where there is no ground truth. There is less noise (random variation of voxel brightness) apparent in the PCA-denoised images than in the competing approaches (e.g. figure 6: livers in the abdominal scans; green arrow in brain #2). Figures 6 and 7 suggest that PCA-denoising retained more image detail than synthetic MRI, which could potentially be due to reduced systematic errors with PCA-denoising, but may also be related to patient motion effects and diffusion anisotropy. In the presence of motion, PCA-denoising appears to have sharper boundaries than synthetic MRI (e.g. figure 6: several vessels visible in liver (blue arrows abdominal axial and abdominal coronal)). Signal intensity in PCA-denoised images better reflects the signal intensity in the noisy image (e.g. figure 6 in kidneys from abdominal axial #2 (green arrow)), which enables taking diffusion directionality into account in this approach of denoising (e.g. figure 6, the extent of ventricles in the brain (blue arrow)). The combined increase in SNR and sharpness allows for better depiction of tissue types (e.g. fascia in the leg image (blue arrow); pancreas in abdominal axial (green arrow) and axial #2 (blue arrow); in abdomen coronal green arrows indicate small hypointense regions; sharper borders between white and grey matter in brain #2 (blue arrow)). In the pancreatic cancer patients (figure 7) the sharper imaging and increased SNR resulted in more conspicuous borders of the organs and tumour (red arrow), particularly visible on the coronal reconstructed images.

When the full dataset was denoised in healthy volunteers, 15, 22, 20, 24, 19 and 4 PCs were selected for the leg, abdomen axial, abdomen axial #2, abdomen coronal, brain and brain #2, respectively. Note that brain #2 only included one image per *b*-value. For the three pancreatic cancer patients, 19, 21 and 21 PCs were selected. PCA-denoising was on average 168 (range 72–645) times faster than bi-exponential synthetic MRI and 37 (22–45) times faster than mono-exponential synthetic MRI *in vivo*, with the biggest gain in larger datasets (table 1 shows timing data).

## 4. Discussion

We have presented a novel PCA-based approach for model-free denoising of multi-*b*-value DW-images. We showed in simulated data that PCA-denoising was able to reduce random noise in DW-images at least as well as model-based techniques, such as synthetic MRI. We have also shown in simulations that PCA-denoising did not add systematic errors across a range of simulated attenuation models, whereas synthetic MRI adds systematic errors that can be substantial, depending on the level of agreement between the true and assumed models. We also show that PCA-denoising works well *in vivo*, in healthy volunteers and patients, and resulted in sharper and less noisy images compared to synthetic MRI. In patients, this can lead to sharper tumour boundaries, which would improve assessments of the tumour extent. These examples also demonstrate sharper tumour edge visualisation, which will be beneficial when determining tumour extent. This may improve tumour delineation for e.g. radiotherapy, where accurately determining tumour extent is crucial. Therefore, we believe that PCA-denoising is a valuable tool for denoising of multi-*b*-value DWI for clinical assessments.

So far, PCA has been applied to anatomical imaging (spatial domain) (Muresan and Parks 2003, Manjón *et al* 2015), multi-echo imaging (quantitative domain) (Bydder and Du 2006), DCE (quantitative domain) (Balvay *et al* 2011) and diffusion tensor imaging (quantitative domain) (Manjón *et al* 2013). In particular, (Pai *et al* 2011) used PCA prior to removing noisy outliers when calculating maximum intensity projection in cardiac DWI. Furthermore, (Chen *et al* 2018) recently utilized diffusion-matched PCA on the magnitude and phase DW data to denoise high-resolution brain diffusion tensor imaging. Spinner *et al* (2018) introduced k-b PCA in IVIM images, which enables higher parallel imaging without adding additional noise.

The approaches cited above in which PCA was applied for DWI mostly focus on brain imaging (Manjón *et al* 2013, Chen *et al* 2018, Spinner *et al* 2018), meet very specific denoising needs (reduce noise for MIP (Pai *et al* 2011), enable higher parallel imaging (Spinner *et al* 2018)) and are evaluated with regard to their effect on diffusion modelling. We set out to develop a generalizable and simple approach for improving image quality for diagnostic purposes that could be easily applied in any anatomy. We have evaluated its performance with this purpose in mind. By only utilizing the quantitative domain (i.e. *b*-value information), we minimize potential blurring of the image. We quantified the improvement of PCA-denoising by comparing it to the ground truth in extensive simulations, and we demonstrate its performance in exemplary clinical data.

For synthetic MRI, the performance strongly depends on how well the denoising model matches the model underlying the data. The data simulated using bi-exponential and stretched-exponential models result in the largest systematic errors. The systematic error introduced by synthetic MRI is less apparent, but still present, when data are simulated using the Kurtosis model. This might be explained by the fact that we have chosen *b*-values typically used for IVIM experiments, whereas the Kurtosis model is more appropriate for capturing effects that appear at higher *b*-values (>1000 s mm^−2^). *In vivo*, the processes contributing to the signal decay are more complex than our current simplified diffusion models, and hence synthetic MRI does probably never fully describe the data.

We showed that for the range of *b*-values used in these simulations, the performance of PCA-denoising is best for intermediate *b*-values (figure 5). Because PCA is a subspace projection technique, the PC vectors implicitly encode some degree of data sharing between the different *b*-value images, and this will tend to be strongest for images with similar *b*-values. Therefore, images that are in the middle of the acquired *b*-value range will have a larger pool of images having relevant data to share from. Hence, if images with a given *b*-value are desired for diagnosis or delineation, it is advisable to acquire *b*-values above and below this to ensure best denoising performance.

We found that reconstructing denoised images with fewer PCs generally resulted in a lower random error (e.g. figures 2(e)–(f)). However, when insufficient PCs were used, the systematic error increased, as the diffusion attenuation signal could not be fully captured. Our method for selecting the optimal number of PCs resulted in the systematic error reaching negligible levels, while the random error was similar to synthetic MRI (figures 2(e), (f) and 4). In contrast, the systematic errors for the synthetic MRI depended strongly on whether the denoising model matched the true model.

Denoising of the *in vivo* images used more PCs (4–24) than the simulations (2–4). There are several reasons why selecting more PCs may be essential *in vivo*. Our *in vivo* data had more *b*-values than the simulations, which might require more components to be described properly. Furthermore, motion can lead to more PCs with coherency. Also, the effect of diffusion directions may have required more PCs to be included. Finally, the actual signal decay *in vivo* may be more complex than in our simulations, resulting in more PCs being required. The latter is especially interesting, as denoising by synthetic MRI would cause systematic errors here.

The PCA-denoised *in vivo* images had visually sharper boundaries than the synthetic MRI and averaged images. We believe that this is due to the fact that motion can be captured and removed by removing latter PCs, as shown in other research on DCE data (Melbourne *et al* 2007). In preliminary simulations (Supplemental Digital Content 3) performed on the downloaded brain dataset (brain #2, Peterson 2016), we investigated how induced motion impacted PCA-denoising. Here, we saw that contrary to averaging and synthetic MRI, the PCA-denoised images did not show blurring in the presence of motion. For large motion, more PCs were required for reconstruction, substantiating our suggestion that motion is taken into account in the PCs. The exact interplay between motion and PCA-denoising is an interesting topic for future research.

Reconstruction times were considerably shorter for PCA-denoising (0.41–7.3 s) compared to synthetic MRI (26.5–937.3 s). We used the ‘pca’ function from the MATLAB statistics toolbox, which uses efficient optimized routines for the required matrix computations that avoid the need for explicit looping. However, for synthetic MRI with least squares fitting, for which we used the ‘fminsearch’ command from MATLAB, it is necessary to explicitly loop over voxels, which results in considerably longer run times. The synthetic MRI was implemented using parallel for loop (‘parfor’ in MATLAB) over four 3.5 GHz cores (Intel Core i7-4771). There are more advanced fitting algorithms available than the fit used (Barbieri *et al* 2016, While 2017, Gurney-Champion *et al* 2018), but these often take significantly longer and still suffer from model-dependency.

The only manual step in the analysis was selecting the threshold level to determine relevant foreground and background voxels. Automated solutions are available for more homogenous data (as done in pre-processing DWI by vendors), but due to the multi-vendor, multi-protocol nature of our data, we decided to manually select this threshold.

There are several limitations to PCA-denoising. PCA-denoising is limited to reconstructing only the acquired *b*-value images whereas synthetic MRI allows for reconstructing any *b*-value image (Blackledge *et al* 2011, Winfield *et al* 2016) However, when synthetic MRI is used to reconstruct images outside the range of acquired *b*-values, noise can be magnified due to the extrapolation of the data. Therefore, synthetic MRI is still often used to generate images at *b*-values close to the acquisition range.

Another limitation is that, in this work, PCA-denoising was optimised for typical IVIM datasets with a large number of *b*-values. However, some research suggests that shorter acquisitions with 4–7 *b*-values can be used for IVIM modelling (Dyvorne *et al* 2014, Gurney-Champion *et al* 2016), where the number of measurements is only equal to, or a little larger than, the number of parameters in the model. When used for denoising, PCA is operating as a data reduction technique, which implies the data dimensionality should be larger than the space onto which it is reduced. Hence, for short acquisitions, PCA might not work as well and further work is needed to evaluate how few *b*-values and/or averages are required for denoising to be feasible. This is also true for synthetic MRI, which, to be able to remove any noise, requires data from at least one *b*-value more than the model’s degrees of freedom.

A limitation of this study is the small sample size of nine subjects that were used for evaluation. However, the examples presented here cover a broad spectrum of data acquisition schemes, all of which PCA-denoising performed well in, highlighting the flexibility of the algorithm. The algorithm is also being shared (Supplemental Digital Content 1) such that readers can test it on their own dataset.

In the patient data, PCA-denoising resulted in sharper and less noisy images than using the alternative denoising approaches. This could greatly benefit any application where accurately determining the boundary and extent of tumours is of importance. This is of particular relevance in radiotherapy applications, where the ability to conform high prescription dose to arbitrary regions, with sharp dose falloff to nearby surrounding tissue, is now commonplace, and so it is necessary that the imaging used to determine this region is of the best quality possible. As high *b*-value images have good contrast in relation to the solid parts of tumours, high *b*-value DW images are of interest for tumour delineation in radiotherapy (Gurney-Champion *et al* 2017, Heerkens *et al* 2017). On the other hand, tumour response may be related to both perfusion and diffusion effects (Li and Padhani 2012, Park *et al* 2014, Hauser *et al* 2014, Xiao *et al* 2015), which can be assessed by acquiring a wide range of *b*-values, including many low ones. We demonstrated that PCA-denoising has potential to greatly improve the quality of the high *b*-value DW images from such IVIM datasets, which can enable more accurate tumour delineation.

In conclusion, multi-*b*-value MRI can greatly benefit from model-free denoising with our PCA-denoising strategy. We showed that the random error is reduced without introducing systematic errors. Also, *in vivo* images denoised by PCA were generally sharper than synthetic MRI or averaging. The technique has great potential for use in applications where knowledge of exact tumour extent is important, for example for diagnosis or in radiotherapy.
